# Anti-IgE therapy versus allergen-specific immunotherapy for food allergy: weighing the pros and cons

**DOI:** 10.3389/fimmu.2025.1617153

**Published:** 2025-07-22

**Authors:** Michael D. Kulis, Jessica R. Humphrey, James W. Krempski, Edwin H. Kim, Johanna M. Smeekens

**Affiliations:** ^1^ Division of Allergy and Immunology, Department of Pediatrics, School of Medicine, University of North Carolina at Chapel Hill, Chapel Hill, NC, United States; ^2^ Curriculum in Toxicology and Environmental Medicine, School of Medicine, University of North Carolina at Chapel Hill, Chapel Hill, NC, United States

**Keywords:** omalizumab, allergy immunotherapy, oral immunotherapy, sublingual immunotherapy, epicutaneous immunotherapy, IgE, desensitization

## Abstract

With the recent FDA approval of the anti-IgE biologic, omalizumab, in 2024 for the treatment of food allergy, it is critical to consider the advantages and disadvantages of anti-IgE and allergen-specific immunotherapies (AITs) to help determine optimal patient care. Several AITs have been studied for food allergy, including oral (OIT), sublingual (SLIT), and epicutaneous immunotherapy (EPIT) with varying degrees of safety and efficacy. There are obvious advantages of treating food allergies with omalizumab, including less frequent administration (every 2 or 4 weeks) compared to the daily dosing of AITs, treating multiple food allergies with one medication, and the potential benefit for comorbid asthma and environmental allergies. However, disadvantages of omalizumab include the requirement for lifelong treatment of a costly biologic that will not induce immunologic tolerance. On the other hand, AITs have been shown to effectively induce desensitization in most individuals and can lead to long-term tolerance or remission in a subset of patients. In this review, we will discuss the pros and cons of omalizumab and AITs and the potential benefit of combining both approaches in young children to achieve immediate increases in reaction threshold while also inducing tolerogenic immunologic responses.

## 1 Introduction

Food allergies are caused by cutaneous, airway, or gastrointestinal allergen exposure prior to the induction of oral tolerance, leading to the production of allergen-specific IgE. Underlying IgE production by allergen-specific B cells are pathogenic Th2a and Tfh cells that secrete Th2-type cytokines, including IL-4 and IL-13 (1–3). IgE binds to its high affinity receptor, FcεRI, on mast cells and basophils, which degranulate upon subsequent allergen ingestion, leading to allergic symptoms (4). Allergic symptoms can be relatively minor, including hives, itching, and gastrointestinal symptoms, or can become life-threatening if anaphylaxis results. Living with food allergies, and the possibility of experiencing anaphylaxis, leads to a significantly impaired quality of life (5).

Although the reasons are unclear, food allergies have increased in prevalence over the last few decades, now impacting an estimated 10% of the population in the United States (6). Researchers have spent the better part of the past 30–40 years developing and testing a variety of treatment approaches for food allergies; however, in most cases the standard-of-care is strict avoidance paired with teaching how to recognize allergic symptoms and prescribing injectable epinephrine to reverse severe reactions (6). One of the first reported proof-of-concept studies for food allergy immunotherapy appeared in 1992 with the use of subcutaneous injections (SCIT) of aqueous peanut extract (7). A larger follow-up clinical trial of peanut SCIT in 1997 demonstrated improvement in peanut-specific immune responses, however, participants experienced repeated allergic reactions to the injections leading to the abandonment of this approach (8). Another potential treatment option for peanut allergy, anti-IgE therapy, was first reported in 2003. Anti-IgE therapy was found to have a dose-response effect and increased reaction thresholds in some subjects (9). These initial reports provided evidence that both allergen-specific and allergen-independent strategies could potentially be viable treatments for food allergy, setting the stage for the next two decades of research.

From approximately 2005-2025, the field of food allergy therapeutics has seen an explosion of clinical trial reports and novel attempts to “desensitize” the Th2-IgE immunologic programming in subjects with food allergies. Several allergen-specific immunotherapies (AIT) have been explored, including oral, sublingual, and epicutaneous immunotherapies (OIT, SLIT, and EPIT, respectively), resulting in dozens of clinical trials (10). Allergen-independent therapies have included anti-IgE therapy and an herbal formula, FAHF-2 (11). A few studies have also examined combining anti-IgE with OIT (12). While these trials have all focused on increasing the reaction threshold in individual subjects, there have been a range of safety concerns and variability in efficacy. Mechanistic studies within these trials have provided invaluable insights into how the immune system is modulated and how durable these changes are. Now, in 2025, there are two FDA-approved products for food allergy, Palforzia (OIT) and omalizumab (anti-IgE) (13). In this review, we will discuss both anti-IgE and AIT, delving into the advantages and disadvantages of each approach. We will conclude with our thoughts on the currently available therapies and our vision for the next iteration of treatment goals in this field.

## 2 Anti-IgE therapies

IgE is a requisite for food allergies, and therefore neutralizing IgE has long been seen as a potential therapeutic strategy. The first anti-human IgE monoclonal antibody tested for food allergy was TNX-901, which is a molecule that blocks the epitope on IgE that binds to FcϵRI (9). Three doses of TNX-901 were evaluated in comparison to placebo, demonstrating that the highest dose administered, 450 mg, every four weeks for four doses significantly increased the amount of peanut required to cause a reaction. These promising results eventually led to the development of another anti-IgE monoclonal antibody, omalizumab, which initially gained FDA approval in 2003 for the treatment of asthma, and later for chronic spontaneous urticaria, and chronic rhinosinusitis. Since then, researchers have used omalizumab as an adjunct therapy for OIT and have shown that a run-in period with omalizumab enables patients to tolerate higher OIT doses (14–16). These observations along with omalizumab’s proposed mechanism of action led to the hypothesis that omalizumab alone may be enough to increase oral food challenge reaction thresholds in a safe and effective manner.

Most recently, the OUtMATCH trial was designed to test omalizumab for treating multi-food allergic participants by biweekly or monthly dosing for 16 weeks (17). Food allergic participants all had double-blind, placebo-controlled food challenge (DBPCFC)-confirmed peanut allergy and at least two other food allergies from the selection of cashew, egg, milk, walnut, hazelnut, and wheat. The primary endpoint was consumption of at least 600 mg of peanut protein during a post-omalizumab DBPCFC, and key secondary endpoints were consumption of at least 1,000 mg of cashew, egg, or milk protein. The data convincingly demonstrated 67% of peanut allergic participants met the primary endpoint, compared to 7% that received placebo. The key secondary endpoints were also met with 41% of cashew-allergic, 67% of egg-allergic, and 66% of milk-allergic participants consuming more than 1,000 mg allergen. Importantly, many subjects were able to tolerate over 4,000 mg of allergen upon oral challenge, demonstrating protection against large quantities of allergen and definitive protection from cross-contamination. As OUtMATCH was a Phase 3 trial, it led to the 2024 FDA approval of omalizumab for food allergies in patients one year of age and older.

**Figure 1:**
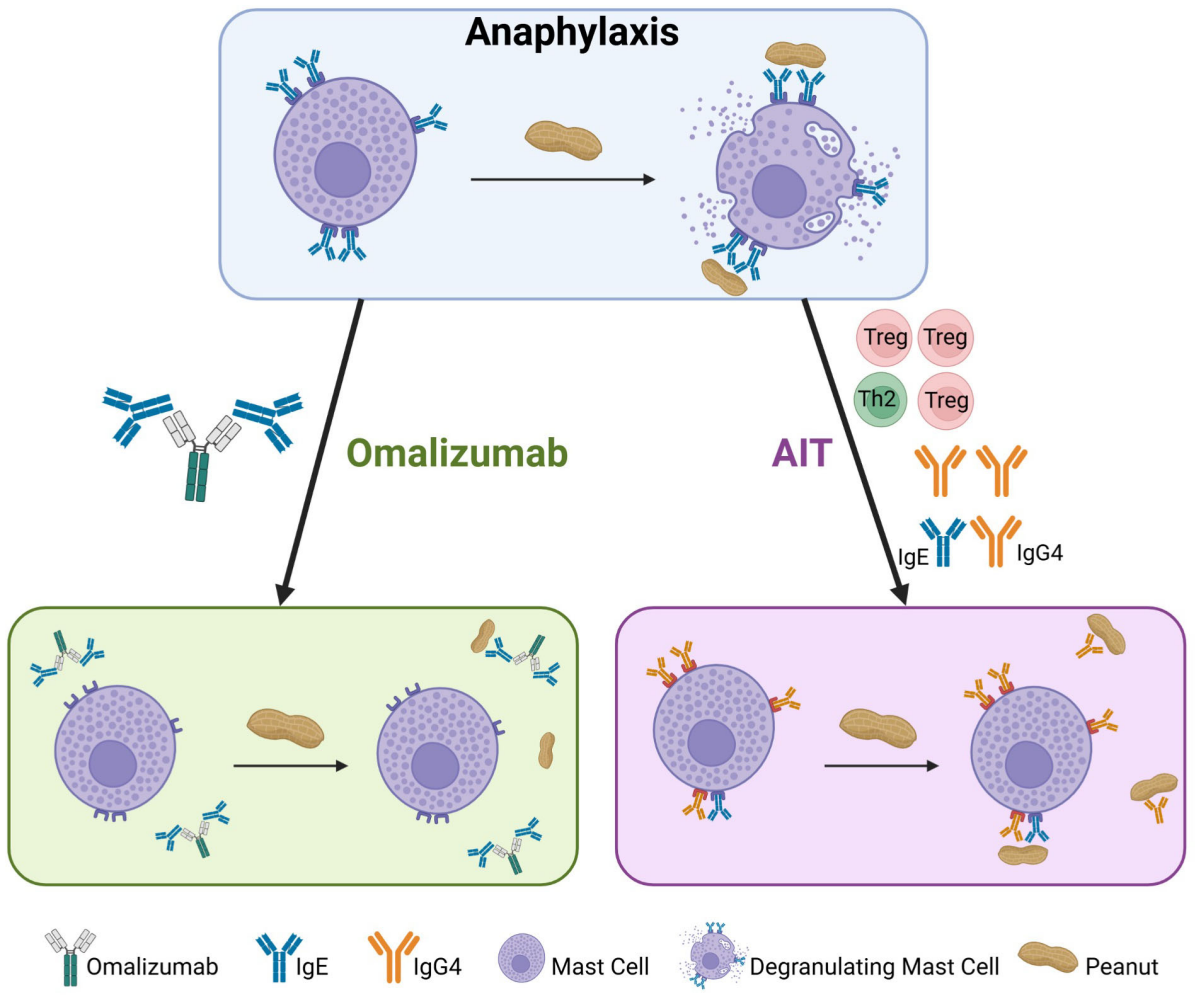
Mechanisms of action for omalizumab and allergen-specific immunotherapy (AIT). In peanut allergic individuals, IgE coats mast cells, which upon peanut exposure, degranulate releasing allergic mediators that cause allergic symptoms (top panel). Omalizumab binds free IgE resulting in absence of peanut-specific IgE on mast cells, which do not degranulate during peanut exposure (left panel). AIT reduces Th2-type cells and cytokines with an increase in Tregs, ultimately leading to decreased peanut-specific IgE and increased peanut-specific IgG4. Upon peanut exposure, mast cells do not degranulate owing to peanut-specific IgG present on mast cells and the neutralization of peanut by IgG4 (right panel). Created in Biorender.

Omalizumab’s mechanism of action results from masking the key epitope required for free circulating IgE to bind to high affinity FcϵRI on mast cells and basophils (**Figure 1**) (18). By preventing IgE from engaging receptors on effector cells, the remaining IgE-bound on mast cells or basophils is eventually diminished, meaning there is limited ability to cross-link IgE and cause degranulation after allergen is encountered. Additionally, omalizumab downregulates the number of FcϵRI on effector cells, further preventing any unbound, circulating IgE from binding to mast cells and basophils (18). However, IgE is constantly produced by long-lived plasma cells in the bone marrow, therefore repopulating circulating IgE, thus requiring repeated omalizumab administrations (19).

## 3 Allergen-specific immunotherapy

Allergen-specific immunotherapy is administered using very small doses of allergen that increase over time to eventually desensitize effector cells and prevent allergic reactions. Over the past two decades, three types of immunotherapy have been investigated for food allergy with different administration routes: oral, sublingual, and epicutaneous immunotherapy (20).

### 3.1 Oral immunotherapy

OIT is administered as a flour derived from an allergenic food, typically mixed in with a food vehicle and ingested daily. Peanut OIT was approved by the FDA in 2020 under the drug name Palforzia, which is a pharmaceutical-grade peanut flour. Preceding FDA approval of a peanut OIT drug product, there were numerous clinical trials of peanut OIT (21–25). One of the first, large, controlled Phase 2 trials, called STOP II, enrolled 99 participants aged 7–15 years who underwent 6 months of OIT or strict avoidance followed by cross-over to active OIT (26). In the first phase, 62% of subjects in the OIT group achieved desensitization with a cumulative tolerated dose of at least 1,400 mg peanut protein compared to 0% of the control group. After cross-over to active OIT for 6 months, 54% of the control group became desensitized. Another trial of high importance, IMPACT, was conducted in preschool aged children 1–4 years old (27). Participants were randomized to peanut OIT (n=96) or placebo (n=50) for ~30 months before undergoing a DBPCFC. 71% of the peanut OIT group, and only 2% of the placebo, achieved desensitization with a cumulative tolerated dose of 5,000 mg. The pivotal Phase 3 trial, PALISADE, evaluated 496 participants aged 4–17 years old (28). Participants consumed peanut OIT or placebo for approximately 12 months then underwent a DBPCFC to assess desensitization. 67% of the peanut OIT group and 4% of placebo achieved desensitization with a cumulative tolerated dose of 1,043 mg peanut protein. Despite this high rate of desensitization, side effects in the OIT group were common, with 11.6% of participants withdrawing from the trial. Another Phase 3 peanut OIT trial, POSEIDON, was conducted in younger children, aged 1–4 years. Participants received peanut OIT (n=98) or placebo OIT (n=48) for 12 months, with 74% of participants in the OIT group meeting the primary endpoint of tolerating a single dose ≥600 mg peanut protein, while 6% of the placebo group met the primary endpoint (29). These results from the PALISADE and POSEIDON trials ultimately led to the FDA approval for Palforzia in patients 1–17 years of age. Several other food-specific OIT trials have been reported with similar efficacy, including egg (30), milk (31), walnut (32), cashew (33), sesame (34), and wheat (35), suggesting this is a promising therapy for a wide range of food allergies.

### 3.2 Epicutaneous immunotherapy

Peanut EPIT is administered as an adhesive patch with an occlusive chamber that contains dried peanut proteins. The proteins are solubilized with moisture from the skin, leading to absorption and presentation to the immune system. EPIT is being developed as the Viaskin patch and has undergone Phase 3 clinical trial testing. In the PEPITES trial, 356 participants aged 4–11 years old (median age 7) were enrolled and randomized to receive peanut EPIT or placebo (36). After 12 months of treatment, DBPCFC were conducted demonstrating that 35.3% of peanut EPIT were responders compared to 13.6% of placebo. Unfortunately, this result did not meet the pre-defined statistical primary endpoint, therefore requiring further studies before FDA approval. In a subsequent Phase 3 trial, EPITOPE, a younger population of participants were enrolled. A total of 362 participants aged 1–3 years old (median age 2.5) were randomized 2:1 to peanut EPIT or placebo, respectively. After 12 months of treatment, 67% in the peanut EPIT group met the primary endpoint for desensitization compared to 33.5% of the placebo group (37). During the open label extension period, 244 participants continued peanut EPIT and completed the 24-month DBPCFC. The additional treatment period led to increased desensitization, with 81% tolerating >1,000 mg peanut protein and 56% tolerating 3,444 mg (38). Although remission has yet to be tested, these trials demonstrate that peanut EPIT can effectively desensitize peanut allergic children, with the highest rate of success in preschool-aged children undergoing multiple years of treatment. Currently there is an ongoing Phase 3 study for peanut EPIT in 4-7-year-old children (NCT05741476), which may ultimately lead to FDA approval. While the clinical trials with Viaskin have focused on peanut allergy, it is presumed that EPIT will be similarly effective for other food allergies, such as milk and tree nuts which have begun early stage clinical testing and preclinical animal studies, respectively (39, 40).

### 3.3 Sublingual immunotherapy

SLIT consists of an allergen extract administered under the tongue. The plausibility of this administration route is based on Langerhans cells in the oral mucosa, which have tolerogenic properties (41, 42). Several clinical trials have evaluated peanut SLIT, while only small studies have been conducted for milk and hazelnut (31, 43). The first reported peanut SLIT trial was conducted as a single-center, randomized, placebo-controlled trial in 18 participants aged 1–11 years old (median 5.2 years). After 12 months, the DBPCFC demonstrated a 20-fold higher threshold for subjects that received peanut SLIT compared to placebo (44). Participants then continued therapy for up to 5 years. During the DBPCFC, 67% consumed at least 750 mg and 25% tolerated 5,000 mg of peanut protein (45). A larger, multi-center trial conducted in 40 older children and adults (median age 15 years) demonstrated that 44 weeks of SLIT led to a 70% response rate in the SLIT group compared to 15% in placebo (46). An open-label peanut SLIT trial was reported in 1–11 year old (mean age 7.1 years) children to assess both desensitization and sustained unresponsiveness. After 48 months of treatment, 70% of subjects tolerated >800 mg peanut protein and 36% tolerated 5,000 mg. Sustained unresponsiveness of at least 22 weeks was demonstrated based on DBPCFC data and statistical modeling (47). Finally, a multi-center, double-blind trial in 1–4 year old children was conducted to assess the efficacy of peanut SLIT for desensitization and remission in preschool aged children (48). After 36 months of SLIT, 60% safely consumed 4,443 mg of peanut protein while 0% of placebo tolerated that amount, clearly demonstrating the desensitization effect of peanut SLIT. Participants then stopped SLIT dosing for 3 months and underwent a follow-up DBPCFC to assess remission. 48% of the peanut SLIT group achieved the remission endpoint compared to 0% of the placebo group. Taken together, peanut SLIT effectively desensitizes the majority of participants and can induce long-lasting tolerance, especially in preschool aged children. Ongoing clinical trials are investigating tree nut SLIT (NCT05521711) and the use of tablet-based peanut SLIT rather than liquid extract (NCT05440643).

### 3.4 Mechanisms of action

The same fundamental mechanisms underlying the efficacy of these AITs involve modulation of IgE, IgG4, IgA, T cells, basophils, and mast cells (**Figure 1**). Desensitization can be monitored by quantifying decreased mast cell activation through a skin prick test (SPT); similarly, suppression of basophils through basophil activation tests (BAT) are also associated with allergen-specific desensitization (49–51). Generally, AIT leads to decreased allergen-specific IgE, increased allergen-specific IgG4 and mucosal IgA after several months or years of treatment (52). Upstream of these immunoglobulin changes, CD4+ T cell function is altered as evidenced by decreased Th2 type cytokines and Th2a cells, with some studies showing evidence of Treg induction and their association with clinical outcome (1, 25, 53).

## 4 Comparisons of anti-IgE and AIT

**Figure 2:**
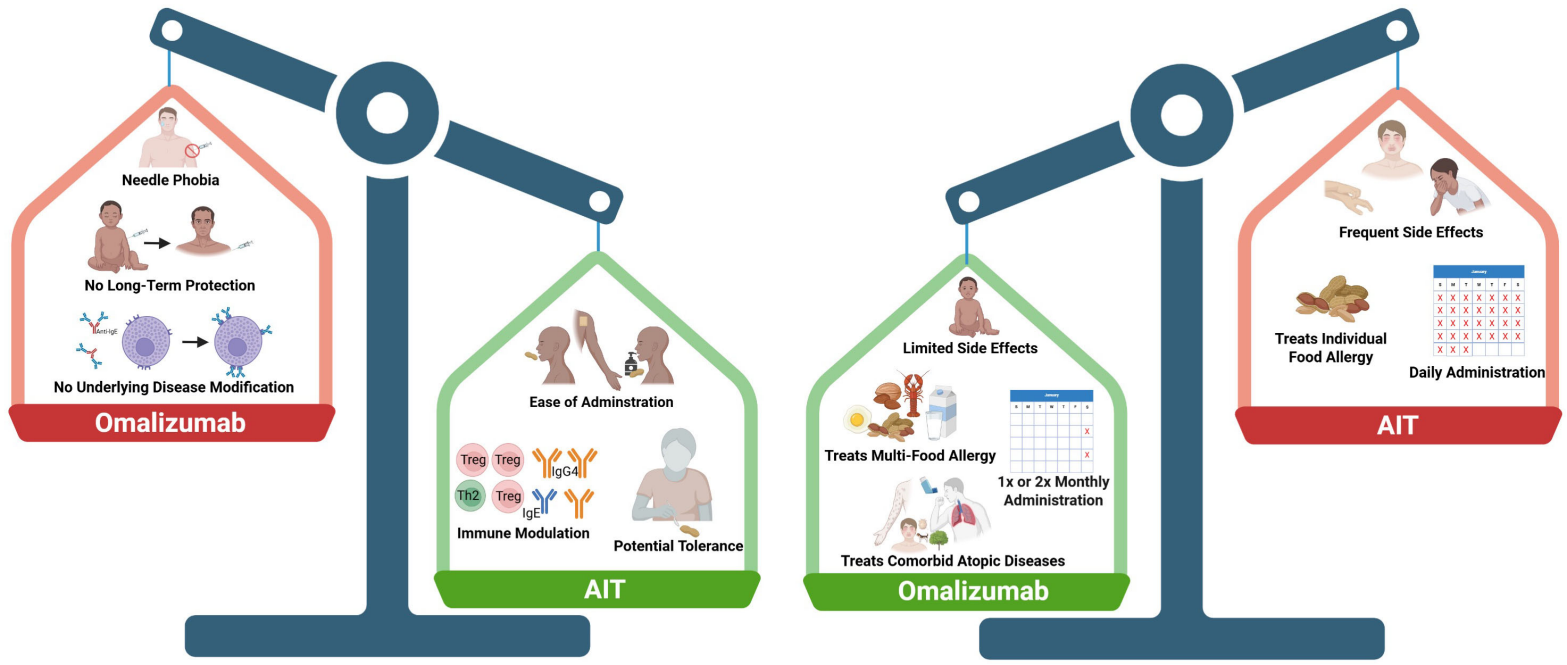
Comparing the pros and cons of omalizumab and allergen-specific immunotherapy (AIT). There are many factors to consider when comparing the two types of therapies, including administration routes and frequency, long-term immune modulation, side effects, and comorbid allergic conditions. The left panel shows potential advantages of AIT (green) compared to omalizumab (red). The right panel shows advantages of omalizumab (green) compared to AIT (red). Created in Biorender.

In this section, we will compare and contrast AIT and anti-IgE therapy for food allergy with a focus on the immunologic effects and how these therapies may be used clinically. While each form of therapy has its advantages and disadvantages, we aim to summarize concepts that are relevant for researchers, providers, and patients (**Figure 2**).

### 4.1 Disease modifying effects

Allergen-specific immunotherapy modulates several compartments of the immune system in patients undergoing treatment. OIT, SLIT, and EPIT universally lead to increased allergen-specific IgG4, which has been shown to inhibit mast cell and basophil degranulation (49). After several months of AIT, a decrease in allergen-specific IgE is often reported, leading to dramatic increases in the IgG4 to IgE ratio. Underlying the changes in immunoglobulins are changes in T cell phenotypes, including decreased Th2a cells and Th2 cytokines, and increased Tregs (1, 25, 51, 54). Mucosal immune responses have also been reported to change during OIT and SLIT with an increase in allergen-specific IgA and IgG4 in saliva (55, 56). Overall, these changes lead to hyporesponsiveness in basophils and mast cells as detected by BAT assays and skin prick tests, respectively (57).

While anti-IgE therapy overlaps with AIT in the effector cell modulation (decreased free IgE and mast cell/basophil suppression), it does not impact allergen-specific T cells or increase IgG4 or IgA. Overall, anti-IgE therapy is not allergen-specific, which has broader benefits, but also disadvantages in terms of long-term durability.

Both OIT and SLIT can induce long-term remission of peanut allergy (also called sustained unresponsiveness) after therapy is discontinued. Remission induction is more frequently reported in young, preschool aged children. For example, the peanut OIT trial, IMPACT, examined the effects of a ~2.5 year OIT regimen followed by a 6 month cessation of OIT. Ultimately, 21% of participants achieved remission and tolerated a peanut food challenge after the 6-month avoidance period. Similarly, in a peanut SLIT trial, participants were assessed for remission after 3 years of peanut SLIT, followed by a 3-month avoidance period. 48% of participants achieved remission. Other studies have demonstrated durable tolerance after stopping AIT for 4–8 weeks (23, 24, 58). Collectively, these data support the concept that AIT, after years of daily treatment, can drive food allergy into remission.

Disease modification, with potential for long-lasting tolerance should be a top consideration for treating food allergic patients, especially those that are younger than 5 years of age where remission is most often observed. Since anti-IgE therapy does not lead to sustained immune modulation, it is only effective as long as the drug is injected, leading to long-term reliance for continued efficacy.

### 4.2 Effects on multi-food allergy and co-morbid atopic conditions

Food allergy is typically accompanied by other atopic conditions, such as asthma, eczema, urticaria, and additional allergies to other foods and environmental triggers. Omalizumab is FDA-approved to treat allergic asthma and chronic urticaria and therefore is an attractive option in patients with these conditions in addition to food allergy. AIT does not have any known effects on co-morbid conditions, as it only modulates the immune response to the single allergen being used in AIT. Likewise, many patients (30-86%) have allergies to more than one food and therefore anti-IgE therapy may be the desired treatment pathway rather than implementing several allergen AITs (59, 60).

### 4.3 Potential side effects

AIT is generally undertaken for several years before food challenges are performed, whereas omalizumab has demonstrated positive clinical effects after only 16 weeks (17). This relatively fast clinical efficacy makes anti-IgE therapy attractive. Additionally, the side effects of omalizumab are mild, with typical adverse events limited to injection site reactions, whereas OIT causes gastrointestinal side effects in the majority of patients (61). These gastrointestinal side effects lead to ~20-30% dropout rates in clinical trials. OIT also has the potential to cause eosinophilic esophagitis (EoE), which is a chronic inflammatory condition of the esophagus and often requires discontinuation of OIT (62, 63). OIT can cause systemic reactions during dosing, whereas SLIT and EPIT have notably less frequent severe side effects, typically oral itching during SLIT and local skin reactions in EPIT (38, 47).

### 4.4 Frequency and routes of administration

Patient burden is an important consideration when comparing AIT to anti-IgE. For individuals with needle phobia, AIT has the advantage of ease of administration through oral or cutaneous routes, whereas anti-IgE must be administered via subcutaneous injection. However, omalizumab is administered at two or four week intervals compared to the daily administration of AIT. Additionally, there are more constraints with AIT, in particular, OIT, which requires patients to avoid exercise and warm showers within 2 hours of dosing and withhold dosing if febrile or having an asthma exacerbation (64). Overall, AIT has a larger time commitment compared to anti-IgE. Depending on insurance coverage, which food allergies require treatment, and availability of these treatments at accessible allergy clinics, AIT and anti-IgE therapy pose a potentially significant financial burden on families.

## 5 Combination therapies/future uses

There have been several studies combining OIT with anti-IgE, with the focus of more safely and rapidly achieving maintenance doses for OIT (14, 15). In these studies, omalizumab treatment is started for several weeks before beginning OIT which allows for reduction in free IgE prior to antigen exposure. This combination therapy has been implemented for a multi-OIT study, with 36 participants receiving omalizumab plus multi-OIT and 12 receiving multi-OIT alone (16). After 36 weeks of treatment, 83% of omalizumab plus multi-OIT subjects passed DBPCFCs for two or more of their allergenic foods, compared to only 33% of the multi-OIT alone group. Furthermore, adverse events associated with OIT were significantly reduced in the omalizumab plus multi-OIT group compared to multi-OIT alone.

## 6 Discussion

The FDA approval of Palforzia and omalizumab for the treatment of peanut and other food allergies has changed the landscape of treatment options for food allergies, creating excitement amongst providers, patients, and researchers alike. With these two options, there are many factors to consider when deciding on appropriate treatments for patients. Shared decision making should be implemented to determine which therapy may be ideal on a case-by-case basis (65). Ultimately these decisions must take into account the allergic status of the patient, including the presence of multiple food allergies, severity of asthma, and other atopic diseases. Since omalizumab may be efficacious for multi-food allergy, asthma, and environmental allergies, patients that fall into this category may benefit more from anti-IgE therapy compared to an allergen-specific immunotherapy. However, omalizumab does not induce long-term tolerance to foods, which poses a significant disadvantage. Clinical trials have demonstrated that peanut OIT induces allergen-specific immune responses, with the potential for remission in a subset of individuals, which would be more beneficial for peanut-allergic individuals without other allergies. Additionally, OIT has the advantage in that the patient is consuming several hundred milligrams of peanut protein daily, providing evidence to themselves and practitioners that they have some level of protection, whereas there are currently no biomarkers to show that omalizumab has resulted in desensitization. Future research should focus on biomarker discovery to predict and monitor both OIT and omalizumab outcomes.

Stage 1 of the OUtMATCH study, which led to the approval of omalizumab for food allergy, required strict allergen avoidance during omalizumab dosing. Stage 2 aims to evaluate omalizumab in combination with OIT, and Stage 3 will determine whether previously allergenic food can be incorporated into the daily diet in the absence of ongoing omalizumab or OIT (66). The data from Stage 2 and 3 will help determine whether incorporation of foods, in the form of OIT or dietary intake, or allergen avoidance is the optimal use of omalizumab. This may be especially important for foods like milk, egg, and wheat, where avoidance during childhood can lead to failure to thrive and inadequate nutrient intake (67).

Despite the promise of having two FDA-approved drugs for food allergy, there is still an unmet need for therapeutics that are highly efficacious and long-lasting, with minimal side effects. Based on what we’ve learned from trials of omalizumab, broadly targeting IgE and IgE-binding effector cells may be a promising path forward. Approaches that could delete or tolerize mast cells and basophils have shown promise in preclinical studies (68–71). Given new information about how IgE is constantly produced throughout the lifespan, targeting memory IgG+ B cells may be beneficial (72). Utilizing existing AIT paradigms but incorporating novel aspects such as Th1-skewing adjuvants (73) or antigens presented on virus-like particles or nanoparticles (72, 74, 75) may induce inhibitory antibodies that would lead to long term protection. While these preclinical therapy approaches are promising, clinical trials will need to be conducted to test their efficacy in humans. While there is room for improvement with current therapy options, we have entered an exciting era of food allergy therapeutics.
